# Using satellite-measured relative humidity for prediction of *Metisa plana*’s population in oil palm plantations: A comparative assessment of regression and artificial neural network models

**DOI:** 10.1371/journal.pone.0223968

**Published:** 2019-10-18

**Authors:** Siti Aisyah Ruslan, Farrah Melissa Muharam, Zed Zulkafli, Dzolkhifli Omar, Muhammad Pilus Zambri

**Affiliations:** 1 Department of Agriculture Technology, Faculty of Agriculture, Universiti Putra Malaysia, Serdang, Selangor, Malaysia; 2 Institute of Plantation Studies, Universiti Putra Malaysia, Serdang, Selangor, Malaysia; 3 Department of Civil Engineering, Faculty of Engineering, Universiti Putra Malaysia, Serdang, Selangor, Malaysia; 4 Department of Plant Protection, Faculty of Agriculture, Universiti Putra Malaysia, Serdang, Selangor, Malaysia; 5 Department of Agronomy and Innovation, TH Plantations Berhad, Kuala Lumpur, Wilayah Persekutuan, Malaysia; Newcastle University, UNITED KINGDOM

## Abstract

*Metisa plana* (Walker) is a leaf defoliating pest that is able to cause staggering economical losses to oil palm cultivation. Considering the economic devastation that the pest could bring, an early warning system to predict its outbreak is crucial. The state of art of satellite technologies are now able to derive environmental factors such as relative humidity (RH) that may influence pest population’s fluctuations in rapid, harmless, and cost-effective manners. This study examined the relationship between the presence of *Metisa plana* at different time lags and remote sensing (RS) derived RH by using statistical and machine learning approaches. *Metisa plana* census data of cumulated larvae instar 1, 2, 3, and 4 were collected biweekly in 2014 and 2015 in an oil palm plantation in Muadzam Shah, Pahang, Malaysia. Relative humidity values derived from Moderate Resolution Imaging Spectroradiometer (MODIS) satellite images were apportioned to 6 time lags; 1 week (T1), 2 weeks (T2), 3 week (T3), 4 weeks (T4), 5 week (T5) and 6 weeks (T6) and paired with the respective census data. Pearson’s correlation was carried out to analyse the relationship between *Metisa plana* and RH at different time lags. Regression analyses and artificial neural network (ANN) were also conducted to develop the best prediction model of *Metisa plana*’s outbreak. The results showed relatively high correlations, positively or negatively, between the presences of *Metisa plana* with RH ranging from 0.46 to 0.99. ANN was found to be superior to regression models with the adjusted coefficient of determination (R^2^) between the actual and predicted *Metisa plana* values ranging from 0.06 to 0.57 versus 0.00 to 0.05. The analysis on the best time lags illustrated that the multiple time lags were more influential on the *Metisa plana* population than the individual time lags. The best *Metisa plana* prediction model was derived from T1, T2 and T3 multiple time lags modelled using the ANN algorithm with R^2^ value of 0.57, errors below 1.14 and accuracies above 93%. Based on the result of this study, the elucidation of *Metisa plana*’s landscape ecology was possible with the utilization of RH as the predictor variable in consideration of the time lag effects of RH on the pest’s population.

## Introduction

Bagworm, a leaf defoliating caterpillar, is among the biggest pest threats in oil palm cultivation. In Malaysia, the most economically devastating species of bagworms for oil palm plantations are *Metisa plana* (Walker), *Pteroma pendula* (Joannis), and *Mahasena corbetti* (Tams). Among these three common species, *Metisa plana* have caused the most detrimental effect in Peninsular Malaysia, judging by the magnitude of its infestations and damages [1] that is resulting from efficient dispersion mechanisms that lead to high reproductive success [2]. A damage of only 10% to 13% could affect the oil yield production by up to 40% [3], or even worse, 50% of the damage brought by the insects’ infestation would reduce approximately 10 t ha^-1^ of fresh fruit bunch (FFB) for two subsequent years [4]. The pest is capable of defoliating the palm leaves to the extent that they could jeopardize the ability of palms to photosynthesize through complete skeletonization of leaves and canopies, eventually suppressing the growth of the palms and reducing yield. The economic importance that the pest has brought to the oil palm industry signifies the urgency to control *Metisa plana* infestation at its earliest, through proper execution of rigorous control methods and mitigation actions such as integrated pest management (IPM) and pesticide applications. The success in performing these actions significantly depends on understanding the pest’s spatial and temporal distributions i.e. its ecological preference. Hence, understanding environmental factors such as the influence of weather variables on the extent of bagworm’s outbreak is essential owing to its adverse influence on insect’s behaviour.

Apart from weather parameters such as temperature and precipitation, relative humidity of which is derived from these two, is also found to have a strong influence of insect population dynamics. The influence of relative humidity on insect development and survivability is especially important during early developmental stages of insects. Given higher levels of relative humidity, faster development of normal embryonic and eggs hatchability for some insects have been reported [5–7]. Nevertheless, on both extreme ends of relative humidity values, eggs hatchability might be compromised. While an insufficient level of humidity is able to hinder the release of larvae from eggshell due to the loss of lubrication and hardened cuticle caused by desiccation [8], increased humidity may cause egg mortality due to drowning and pathogen infection [8–11]. Additionally, at excessive humidity, entomopathogenic fungal infections among insects and their larvae will increase [12–14]. In a bagworm related study, Sajap and Siburat [15] demonstrated that a high level of relative humidity was positively correlated with the infections of fungus in bagworm. Since the relative humidity was higher on the middle and the lower tree canopies than at the upper level, a higher number of infected bagworms were found in the higher canopies. Fargues and Luz [16] further emphasized the time of exposure towards the favourable humidity as equivalently essential as the level of relative humidity. A longer exposure towards favourable humidity would increase the potential of fungal infections on insects. The authors stressed that given the combination of temperature and relative humidity, the latter can take precedent due to high cases of fungal infections on insects that were observed within the unfavourable ranges of temperature.

In addition to duration, the immediacy of impacts of external abiotic factors towards insect population dynamics may vary; they can either be immediate or delayed. Studies by Wood and Foot [17], Karpakakunjaram et al. [18] and Intachat et al. [19], for instance, have reported the effects of time lags associated with *Aglenchus costatus* Meyl, acridid grasshopper and geometroid moths population, respectively, up to months. Generally, elucidating the immediacy of insects on their population dynamic’s reaction towards their environmental factors is crucial as it can assist to the development of a more precise and accurate prediction model.

The current practice to elucidate the environmental aspects that possibly affect the insect pest outbreaks are still leaning towards the conventional approaches that typically utilizes a single or networking of weather stations. Often, they are sparsely distributed due to limitations such as cost and topography. On the other hand, weather data such as temperature, rainfall, and relative humidity are currently possible to be obtained from satellite measurements in a denser spatial distribution in comparison to the weather stations, which often require data interpolation for a comprehensive spatial quantification. Remotely sensed spectral measurements offer the possibility to acquire weather variables that triggers insect pest outbreaks in rapid, harmless, and cost-effective manners. While many researches have evaluated the utilization of remotely sensed temperature and rainfall for characterizing pest populations at the landscape level [20–24], little attention has been paid into utilizing RS derived RH for the same goal.

Meanwhile, ANN is a sophisticated system whose working principle imitates the way human brain process information i.e. through pattern recognition and relationship determination. Unlike computer algorithms that process information through programming, ANN conduct information processing and knowledge gathering through experience i.e. learning and training of data. ANN has the ability to produce prediction model with higher accuracy compared to regression analysis [25–28], as its advanced mechanism allows it to excel in capturing nonlinear relationships, whereas regression models requires assumption of linearity. ANN has been used to predict the occurrences of pests’ incidences in several studies such as the population density of cotton pest (*Thrips tabaci*) [29], the effect rainfall and temperature to the establishment of aphid (*Myzus persicae*, *Brevicoryne brassicae*, *Aphis gossypii and Erisoma lanigerum*) and mealybug (*Planococcus citri*) [30], the occurrences of paddy stem borer (*Scirpophaga incertulas*) [31], and the population density of Diamondback Moth (DBM) along with its parasitoids [32]. It is notable that neither of these studies considered lag effects in their predictive model development.

Considering the potential devastating impacts of *Metisa plana* towards the oil palm industry in Malaysia and the importance of the application of technologies in controlling them, the objective of this paper is to examine the relationship between the presence of *Metisa plana* at different time lags and RS derived RH, and to evaluate the applicability of statistical and data mining method to model the presence of *Metisa plana* using RH. This is the first application of its kind and the results will produce prediction models that is able to predict the outbreak of *Metisa plana* using RH as the input factor.

## Methods

### Study area

**Fig 1:**
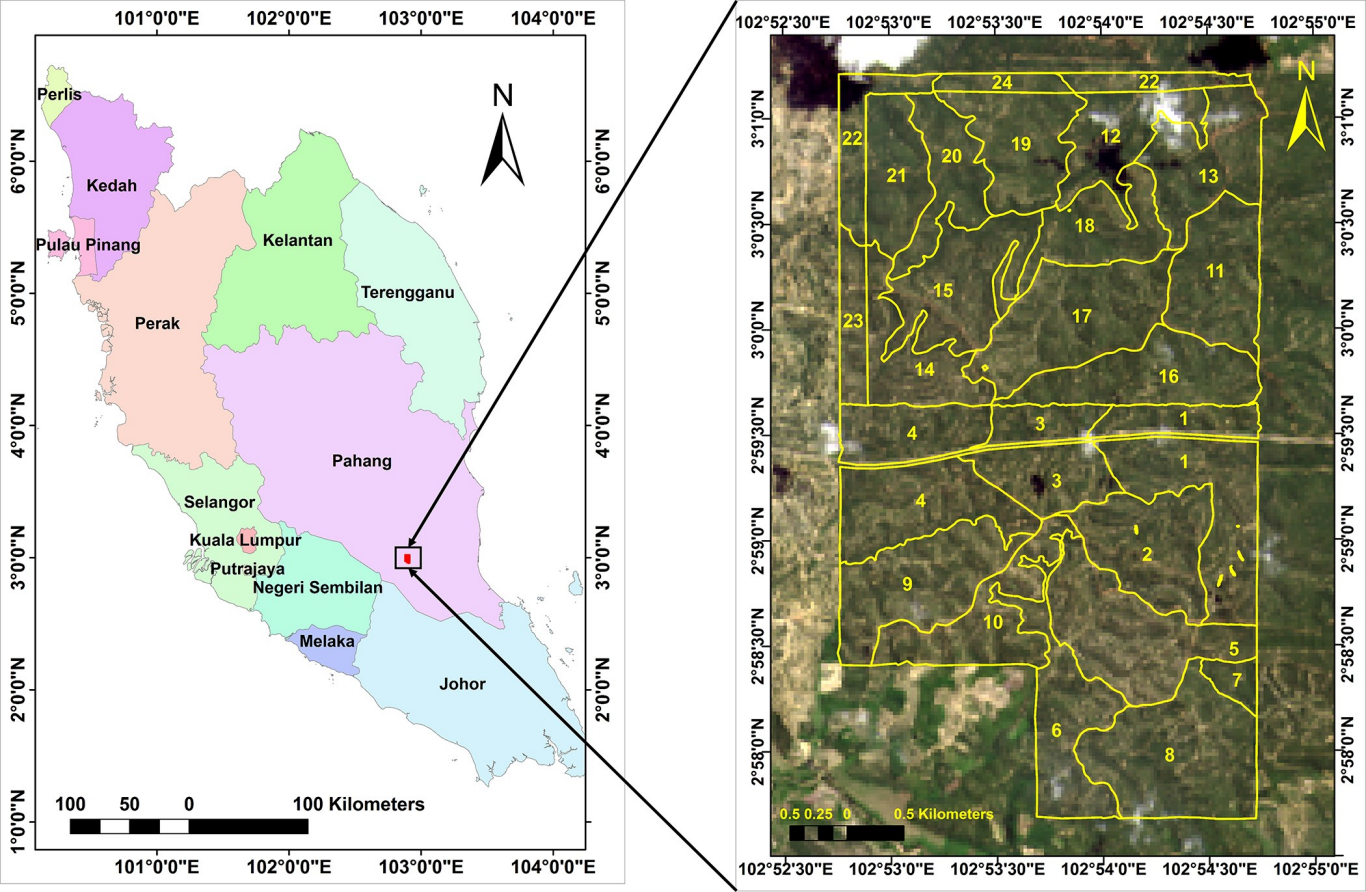
Study area located in Tabung Haji Plantation Berhad’s estate in Muadzam Shah, Pahang, Malaysia (Landsat-8 OLI image courtesy of the U.S. Geological Survey).

This study was conducted in a 2000-ha oil palm estate belongs to Tabung Haji Plantation Berhad in Sungai Mengah, Muadzam Shah, located at 2*°* 57’ 30" N, 102° 53’ 0" E to 3° 1’ 0" N, 102° 53’ 0" E in the state of Pahang, Malaysia (Fig 1). The estate has 26 blocks that are divided into two main divisions which are division A (10 blocks) and B (16 blocks). The ages of oil palm in the study area range from 10 to 20 years old while the bagworm infestation level range from zero to mild infestations i.e. 0 to 32 bagworms per frond. The plantation incorporates IPM with pesticides being applied through trunk injection when the bagworm infestation is above the economic threshold level (>10 per frond). The study area is characterized by temperature ranging from 24°C to 35°C and average annual rainfall ranging from 1900 mm to 2500 mm.

### Data collection

#### Bagworm census data

The census data were collected biweekly over the period of 2014 and 2015, resulting in 24 census cycles annually. The census was conducted according to the standard practice of the plantation management where for each cycle, 25 random palms were selected: 5 palms from each cardinal direction i.e. the north, south, east, west and center of the block. Frond number 17 was destructively pruned for each of the chosen palm as it is considered to represent oil palm crown as a whole [33] and bagworm larvae of instar 1, 2, 3, and 4 from this frond were collected and counted. Only bagworm of these instar level were considered in this study due to the fact that bagworm larvae at these stages bring the most destruction to oil palm owing to the high level of leaf consumption [34]. The total bagworm counts in these 25 palms were then averaged to obtain an average bagworm number per palm per block.

#### Remotely sensed derived relative humidity

The computation of RH was performed according to algorithm described and modified for Malaysia’s utilization by Peng et al. [35]. RH is the ratio of vapour pressure (*e*) and saturation vapour pressure (*e*_*s*_) and can be computed according to Eq 1A, 1B, and 1C:

$$RH=(e/e_{s})\times 100$$ (1A)

Where

e=Q×Pa/0.622 (1B)

$$e_{s}=611\;\exp\;(\frac{17.27\;Ta}{237.3+Ta})$$ (1C)

Where, *RH* stands for relative humidity, *e* stands for vapour pressure, *e*_*s*_ represents saturation vapour pressure, *Ta* denotes surface air temperature, *Pa* signifies air pressure and *Q* represents specific humidity.

The estimation of vapour pressure (*e*) was dependent on air pressure (*Pa*) and specific humidity (*Q*) that was computed from precipitable water vapour (*PW*). Their relationship is described in Eq 2A and 2B. The computation of saturation vapour pressure (*e*_*s*_), on the other hand was dependent on surface air temperature (*Ta*)

$$Q=0.001\times (-0.0762{PW}^{2}+1.753PW+12.405)$$ (2A)

While

PW=(α−(Tobs)/β)2 (2B)

Where, the acquisition of precipitable water vapour (*PW*) was done through the retrieval of atmospheric water content by channel ratio technique that utilizes near infrared (NIR) channels using NIR bands. Among 36 bands of MODIS platform, 5 of them are NIR bands: 2 (0.865μ*m*), 5 (1.24μ*m*), 17 (0.905μ*m*), 18 (0.936μ*m*), and 19 (0.940μ*m*) in which band 2 and 5 are atmosphere window bands while band 17, 18, and 19 are absorption bands that were represented as observed transmittance (*Tobs*) in Eq 2B. The algorithm of the computation of *Tobs* was provided by Kaufman and Gao [36] as the following (Eq 3A, 3B and 3C):

TobsB17(0.905μm)=ρ*(0.905μm)/ρ*(0.865μm) (3A)

TobsB18(0.936μm)=ρ*(0.936μm)/ρ*(0.865μm) (3B)

TobsB19(0.940μm)=ρ*(0.940μm)/[C1ρ*(0.865μm)+C2(1.24μm)] (3C)

Where *Tobs* B17, *Tobs* B18, and *Tobs* B19 stands for the observed transmittance for band 17, band 18, and band 19, respectively (Eq 4A, 4B, 4C). *α* and *β* were the observed transmittance coefficients that was different for each *Tobs* and was provided for Malaysia region by Peng et al. [34] as follows:

TobsB17(0.905μm),α=0.025,β=0.30; (4A)

TobsB18(0.936μm),α=0.056,β=0.60; (4B)

TobsB19(0.940μm),α=0.120,β=0.651 (4C)

It is also worth noting that *ρ* * represents the apparent reflectance which is a ratio between the radiance at the sensor and solar radiance at the top of atmosphere for the specified channel: *C*1 = 0.8 and *C*2 = 0.2 [27].

Since derived water vapour (*PW*) values were different for all three channels, a mean water vapour was obtained using Eq 5.

PW=f1PW1+f2PW2+f3PW3 (5)

Where, *PW*1, *PW*2, *PW*3 are water vapour derived from channel 0.905μ*m*, 0.936μ*m*, 0.940μ*m* or band 17, 18, and 19, respectively. *f* is the corresponding weight of water vapour values of each channels for Malaysia region: *f*1 = 0.36, *f*2 = 0.24, and *f*3 = 0.40.

Finally, the air pressure (*Pa*) used in Eq 1B was obtained using algorithm provided by Peng et al. [35] as shown below (Eq 6):

Pa=1013.3−0.1038H (6)

Where *H* stands for elevation obtained from data provided by shuttle Radar Topography Mission (SRTM) version 3.0 Global 1 arc second Data.

According to Eqs 1 to 6, two sets of MODIS images observed from the Terra and Aqua satellites were required. The first image was MOD07 for the acquisition of surface air temperature (*Ta*) data used in Eq 1C, while the second image was MOD021KM for the acquisition of 5 NIR bands used to compute precipitable water vapour (*PW*). Both of their temporal resolutions were 1 day with spatial resolution of the first product being 5 km and second product being 1 km.

Hence, a total of 444 and 443 of the images for 2014 and 2015, respectively, were utilized in performing the RH derivation. The images were first downloaded from the United States Geological Survey (USGS) Global Visualization Viewer (GloVis) website (https://glovis.usgs.gov/). Further, the required band layers were extracted from each images; surface air temperature layers from the MOD07 images, while layers of NIR bands from the MOD021KM images. All of the layer spatial projections were set to WGS 1984 UTM Zone 47 N and the algorithm was applied to the layers to compute RH images. Finally, all of the images were subset according to the study area.

#### Data extraction

Prior to data extraction, the RH layers pixel size were resampled by nearest neighbour from 1 km to 250 m. The area-weighted mean of RH values for each blocks were then calculated according to Eq 7. Next, the RH values were extracted and paired with the average number of bagworms per palm for each block of each census cycle at 6 different time lags; 1 week (T1), 2 weeks (T2), 3 week (T3), 4 weeks (T4), 5 week (T5) and 6 weeks (T6) prior to the census date. The time lag analysis was carried out based on the hypothesis that the response of insect population towards the effect of abiotic factors in some cases may not be immediate as demonstrated by Wood and Foot [17], Karpakakunjaram et al. [18] and Intachat et al. [19]. Besides, the knowledge of insects responding towards the environmental fluctuation ahead of time is also important in developing a forewarning system of insect outbreak and provide the opportunity for a better and more efficient mitigation program.

$$Area\;weighted\;mean=\frac{\sum \left(pixel\;area\;within\;a\;block\times pixel\;values\;within\;a\;block\right)}{total\;area\;of\;each\;block}$$ (7)

### Data analysis

#### Regression analysis

The relationship between the average bagworm per palm per block with the area-weighted mean of RH of each block at time lag T1, T2, T3, T4, T5, and T6 was determined by Pearson’s correlation coefficient (r). The analysis was later followed by single and multiple regression analyses, linear or polynomial, to measure the magnitude of influence that RH possessed over the *M*. *plana* number. In order to determine the best time lags to be used as the model input, the RH dataset was tested according to these different configurations as follows: (i) individual time-lags i.e. T1, T2, T3, T4, T5 or T6, (ii) divided into two classes of multiple time lags: week 1, 2 and 3 (T1, T2 and T3) or week 4, 5 and 6 (T4, T5 and T6), and (iii) individual time-lags as selected through stepwise selection criteria method. For configuration (iii), among all the time lags analysed, T3, T4 and T5 and T6 were selected for linear regression while T1 and T6 were chosen for polynomial regression. All the statistical analysis was carried out by using statistical analysis software SAS version 9.2 (SAS Institute Inc., Cary, North Carolina, USA).

#### Artificial neural network (ANN)

Three main layers characterize the architecture of neural network: the input, output, and hidden layers. The input layer represents the independent variables, the output layer represents the dependent variables, and the hidden layer represents the relationship developed between input and output variables. The relationship between the input and the output variables will be established by the hidden layers through the adjustment of weightage that is performed iteratively by the training network. In each training, an estimated output will be generated and compared to the actual output values to produce an error term. This error will be used in the network training and the iterative process will be conducted until the minimum error is achieved [37].

In this study, multilayer perceptron ANN was used to further analyse the relationship between dependent and independent parameters to construct the prediction model for the outbreak of *Metisa plana*. The analysis was conducted by using the Alyuda Neurointelligence 2.2 (Alyuda Research LLC, Cupertino, CA). Datasets containing missing values, as well as data anomalies and outliers were excluded before the neural network analysis. All data were then divided into 3 datasets; i) network training dataset which accounted for 60% of the data, i.e. n = 170 ii) network testing dataset which accounted for 20% of the data, i.e. n = 56 and iii) network validation dataset which accounted for remaining dataset, i.e. n = 56. All of the input data were scaled into numbers between -1 to 1 prior to the ANN processing in the next step.

Further, the RH dataset was tested according to the same configurations as in the regression analysis. However, for configuration (iii), forward stepwise feature selection method was conducted where the feature mask with the best fitness value was chosen. Through the feature selection procedure, four time lags were chosen as the input layer i.e. T1, T3, T4 and T6. Prior to the training of the data, network architecture for each input layer was determined as follows: for the (i) input layer, the network architecture was 1-*x*-1, while for the (ii) input layers, a different architecture was assigned i.e. 3-*x*-1 and finally, the (iii) input layer was represented by 4-*x*-1 architecture. The number of hidden layers or *x* for the different input layers were later determined through the best fitness value of the network architecture. The logistic (sigmoid) function was selected as the activation function connecting the hidden and output layers. Network trainings were then performed upon the training dataset using the quick propagation (QP) algorithm. This ANN algorithm was selected since QP is a modification of back-propagation algorithm making it faster than the standard incremental back-propagation, which would be efficient in processing large amount of data in this study [38]. In the training process, the data fed to the network was analyzed and the weights were adjusted according to their influence towards the dependent variables in a series of iterations. The weights were fine-tuned through the validation process of neural network where the number of hidden units were determined and the declination of predictive ability of the neural network was detected. Finally, the output were back transformed to the input data scale prior to the evaluation metrics.

#### Evaluation metrics

The accuracy of predictions for the regression and ANN models were then evaluated and compared by using the adjusted R^2^ values between the actual and predicted *M*. *plana* number. In evaluating the quality of the ANN predictions, two methods were employed in this study which were the absolute error (Eq 8) [39] and testing accuracy (Eq 9) of the training models. The absolute error is the average value of the absolute difference between the predicted and original or actual values, whereby smaller error values indicate a better trained network. On the other hand, the accuracy of prediction models in this study used the min-max accuracy by dividing the minimum average value with the maximum average value among the averaged prediction and actual values and multiplying it by 100 [40]. The minimum-maximum accuracy indicates the deviation of the predicted values from the actual values where the perfect accuracy is 100%.

$$Absolute\;error=\bar{\left|(Predicted\;Values-Actual\;Values)\right|}$$ (8)

Where absolute error represents absolute error for training, validation and testing datasets.

$$Accuracy=Average\;\left[\frac{\min\;\left(actual,predicted\right)}{\max\;\left(actual,predicted\right)}\right]\times 100$$ (9)

## Results

### Relationship between RH and *Metisa plana*

Table 1 presents the correlation results between *Metisa plana* biweekly census and RH of 6 different time lags (T1, T2, T3, T4, T5, and T6). Generally, Pearson’s correlation analysis showed relatively high correlations, positively or negatively, between the presences of *Metisa plana* with RH, ranging from 0.46 to 0.99. Consequently, these values suggested an absence of a linear relationship between the presence of *Metisa plana* and RH. Among 31 correlation coefficient values obtained, 18 of them were negative and 13 were positive. Most of the negative correlations were found in time lag T3 with 6 occurrences. This was followed by T6 with 4 occurrences, and T4 and T5 with 3 occurrences. The least negative correlation frequencies were found in T2 with 2 occurrences. No negative correlation was found in T1. On the other hand, most of the positive correlations were found in T1 with 4 occurrences. This was followed by time lag T4 with 3 occurrences and T2 and T3 with 2 occurrences. The least frequency was found in T5 and T6 with 1 occurrence each.

**Table 1:** Pearson’s correlation between 8-days average relative humidity (RH) with the average number of *Metisa plana* in 2014 and 2015.

**2014**						
**Census cycle**	**T6**	**T5**	**T4**	**T3**	**T2**	**T1**
Cycle 6				-0.79****		0.52*
Cycle 7		-0.74****		0.63**		
Cycle 8						0.91***
Cycle 9					0.65**	
Cycle 12			-0.49*	-0.48*		
Cycle 13	-0.59**			-0.46*		
**2015**						
**Census cycle**	**T6**	**T5**	**T4**	**T3**	**T2**	**T1**
Cycle 1	-0.45*		0.73*	-0.49*		0.46*
Cycle 8	-0.57**	-0.47*	-0.43*		0.51*	
Cycle 9	-0.99****	-0.99****	0.96**	-0.96**	-0.95**	0.97**
Cycle 13	0.67*		-0.68*		-0.68*	
Cycle 19	0.99****	0.82**	0.97****	-0.99****		
Cycle 24				0.97*		

*0.05

**0.01

***0.001

****0.0001 significant levels (P-value). T1, T2, T3, T4, T5 and T6 denotes 1 week, 2 weeks, 3 week, 4 week, 5 week and 6 weeks prior to the census date, respectively. Non-significant cycles were excluded from the table.

The highest significant level (*α* = 0.0001) of correlations was found more in negative correlations as opposed to positive correlations. Among the highest level of negative correlations, two of them were in each time lag T3 and T5. Negative correlations with the highest level of significance occurred at relative humidity within the range of 47% to 64%. Conversely, the positive correlations with the highest level of significance occurred at relative humidity within a narrower range than negative correlations from 64% to 70%.

### Impact of time lag effects on regression analyses and ANN

*Metisa plana* prediction models were generated using linear and polynomial regression models, for a single or multiple RH values (Tables 2 and 3), and ANN (Table 3 and 4). An overall result demonstrated that the highest adjusted R^2^ values were obtained through the ANN i.e. 0.57 and 0.56, both for multiple time-lags. Regardless of the time lags, the linear regressions, either single or multiple, consistently produced models with the lowest adjusted R^2^ that was ranging from 0.00 to 0.05 (Table 3). On the other hand, a distinct pattern was observed for the ANN: high adjusted R^2^ values for models with multiple time lags (0.45 to 0.57) and much lower adjusted R^2^ models utilizing individual time lags (0.08 to 0.31).

**Table 2:** Multiple linear and polynomial regression equation between *M*. *plana* number and relative humidity (RH) at different time lags.

Predictor variables	Linear regression	Polynomial regression
RH at T1	*M*. *plana* number = 15.81–0.23(T1)	*M*. *plana* number = 55.45–1.62(T1) + 0.01(T1^2^)
RH at T2	*M*. *plana* number = 12.47–0.18(T2)	*M*. *plana* number = 58.83–1.92(T2) + 0.02(T2^2^)
RH at T3	*M*. *plana* number = 6.99–0.08(T3)	*M*. *plana* number = 41.42–1.23 (T3) + 0.01(T3^2^)
RH at T4	*M*. *plana* number = 8.47–0.11(T4)	*M*. *plana* number = 47.46–1.42 (T4) + 0.01(T4^2^)
RH at T5	*M*. *plana* number = 5.90–0.07(T5)	*M*. *plana* number = 62.81–2.01 (T5) + 0.02(T5^2^)
RH at T6	*M*. *plana* number = 12.85–0.18(T6)	*M*. *plana* number = 60.82–1.95 (T6) + 0.02(T6^2^)
RH at T1, T2, T3	*M*. *plana* number = 9.48–0.12(T1)	*M*. *plana* number = 9.48–0.12(T1)
RH at T4, T5, T6	*M*. *plana* number = 13.86–0.24(T6) + 0.13(T4)– 0.08(T5)	*M*. *plana* number = 57.40–1.91(T6) + 0.02(T6^2^)
RH at T6, T5, T4, T3 for linear RH at T6, T1 for polynomial	*M*. *plana* number = 16.73–0.29(T6)– 0.16(T5) + 0.11(T4) + 0.09(T3)	*M*. *plana* number = 65.18–1.97(T6) + 0.02(T6^2^)– 0.13(T1)

T1, T2 and onwards denotes RH at week 1, week 2 and so forth prior to the census date, respectively.

**Table 3:** Comparison of adjusted R2 values obtained from linear regression, polynomial regression and artificial neural network (ANN) between the actual and predicted *M*. *plana* number.

Predictor variables	Linear regression	Polynomial regression	ANN
RH at T1	0.05	0.05	0.10
RH at T2	0.00	0.00	0.11
RH at T3	0.00	0.00	0.06
RH at T4	0.00	0.00	0.16
RH at T5	0.02	0.04	0.31
RH at T6	0.00	0.01	0.08
RH at T1, T2, T3	0.03	0.03	0.57
RH at T4, T5, T6	0.00	0.00	0.56
RH at T1, T2, T3, T4, T5, T6	0.01 (T6, T5, T4, T3)	0.01 (T6, T1)	0.45 (T1, T3, T4, T6)

T1, T2 and onwards denotes RH at week 1, week 2 and so forth prior to the census date, respectively.

**Table 4:** ANN error and accuracy terms for different time lags.

Predictor variables	Network architecture	Training absolute error	Validation absolute error	Testing absolute error	Training accuracy	Validation accuracy	Testing accuracy
RH at T1	[1–4–1]	1.51	1.61	1.69	81.20%	78.24%	68.16%
RH at T2	[1–5–1]	0.14	0.17	0.16	99.80%	99.20%	99.80%
RH at T3	[1–2–1]	1.68	1.60	1.67	96.97%	93.53%	88.02%
RH at T4	[1–4–1]	1.55	1.80	1.63	98.22%	83.10%	84.60%
RH at T5	[1–7–1]	1.25	1.47	1.74	97.44%	80.79%	95.95%
RH at T6	[1–7–1]	1.45	1.63	1.75	97.57%	78.30%	68.84%
RH at T1, T2, T3	[3–8–1]	0.56	1.14	1.06	99.56%	93.63%	95.29%
RH at T4, T5, T6	[3–7–1]	0.94	1.35	1.54	93.45%	87.02%	79.40%
RH at T1, T3, T4, T6	[4–9–1]	0.73	1.61	1.18	98.23%	81.34%	89.57%

T1, T2 and onwards denotes RH at week 1, week 2 and so forth prior to the census date, respectively.

Table 4 tabulates the performance of the trained QP algorithm. For the individual time lags, only RH at T2 produced training, validation and testing error below than 1. Indeed, the error terms for this time lag was the lowest among all the models. Multiple time lags, either RH at T1, T2 and T3, RH at T4, T5 and T6 or RH at T1, T3, T4 and T6 similarly had error terms below 1, despite the validation and testing errors were almost one fold of the training error. Other remaining individual time lags had noticeably larger error terms, ranging from 1.25 to 1.68 for the training, 1.47 to 1.80 for the validation and 1.63 to 1.75 for the testing. A considerably different observation could be made for the accuracy terms, whereby there was no clear pattern could be associated with the time lags. While all time lags had a very high training accuracy that was above 90% (except for RT at T1), only RH at T2 and RH at T1, T2 and T3 had both validation and testing accuracies above 90%.

## Discussion

### Relationship between RH with *Metisa plana*’s population

The presence of *Metisa plana* were found to be positively and negatively correlated with both relatively low and high level of RH which suggested that correlation analysis was not able to determine the optimum values of RH that favour *Metisa plana*’s population. All in all, the correlated average relative humidity with the average number of *Metisa plana*’s were recorded to range from 47% to 71%. These correlation results also suggested that the effect of individual, different time lags of RH towards the presence of *Metisa plana* is not pronounced, as the correlation occurrences between RH of different time lags and the presence of *Metisa plana* were more or less the same frequency i.e. no distinct individual time lag was observed to have a significantly more correlation than the other. The low adjusted R^2^ values for the regression models, along with the high adjusted R^2^ and accuracies for the ANN also have confirmed that the relationship between RH and *Metisa plana* was non-linear.

### Modeling algorithms

#### Regression versus ANN

The fact that the ANN models was found to produce higher adjusted R^2^ values than the regression models suggest that the former caters non-linearly and non-normally distributed datasets as opposed to the latter. The regression analysis, for instance, essentially predicts the relationship between dependent (y) and independent (X_1_, X_2_… X_*k*_) variables by assuming that their associations are linear (Eq 10). This include the polynomial regression; the model still assumes the linearity of the relationships when predicting the associations of dependent and independent variables despite it being used to fit a non-linear data into least squares regression (Eq 11).

y=a+β1X1+β2X2…βkXk (10)

y=a+β1X+β2X2+β3X3…βkXk (11)

Whereas *y* represents dependent variables i.e. bagworm numbers and *X* represents independent variables i.e. RH, *a* represents initial intercepts and *β* represents partial regression coefficients of variable*X*.

As a result, this principle limits the ability of regression analysis, either linear or polynomial, to predict non-linear relationships efficiently. The ANN, in contrast, is more superior in assessing large datasets with multivariate interactions and complex patterns [41, 42]. This can be attributed to its ability to self-learn the data and produce models through weight adjustment without preliminary assumptions of the datasets linearity.

In comparing among the ANN architectures, the 3-8-1 architecture with RH T1, T2 and T3 input layer was found to be able to predict the presence of *Metisa plana* with high accuracy and the highest adjusted R^2^ value. Despite the lowest error and highest accuracy terms were produced with the RH at T2 layer with 1-5-1 network architecture, the adjusted R^2^ value for this model was too low i.e. 0.11. A close inspection found that the model failed to predict extreme values of *M*. *plana* at both ends and hence implies a low reliability. Finally, it was noticeable that the absolute error of the best training models utilizing multiple time lags portrayed an almost one-fold increase during the validation and testing phases (from 0.56 to 1.14 and 1.06, and from 0.94 to 1.35 and 1.54, respectively). The amplification of the absolute error was contributed by the overfitting of the training model; likely from the combination of high iteration number and small size of training datasets [43]. Nonetheless, evaluating from the magnitude of the errors, the overfitting could be considered inconsequential and thus, the prediction model was stable and reliable.

#### Time lag effects on modelling algorithms

The effects of time lag was pronounced during the ANN analyses, which generally suggested that multiple time lags could produce high adjusted R^2^ values and acceptable testing accuracies (more than 79%). Individual time lags, on the other hand, resulted in models with unacceptable values of adjusted R^2^ and relatively higher errors than the multiple time lags, despite high overall accuracies. This suggests that, a duration of a week, regardless at any time lags, was insufficient to evaluate the impacts of RH on *Metisa plana*’s population dynamic. In contrast, better accuracies of the multiple time lags models signified the importance to consider the continuity of the RH fluctuations over a longer period towards insects’ population, especially in the more recent weeks. This was manifested through the datasets from the T1, T2 and T3 time lags that were able to generate the highest adjusted R^2^ value and accuracies i.e. above 95%. The results suggest RH fluctuations at the time lag T1, T2 and T3 are more influencing to the insect’s life cycle than at any other time lags. Given the focus of this study that was to predict *Metisa plana*’s larvae of instar level one to four, one to three weeks prior to these stages marks the reproductive stages of *Metisa plana*. This is in alignment with the importance of RH in regulating the efficiency of insects’ reproduction. The optimum level of RH for eggs hatchability is crucial due to the need of lubrication and soft cuticle tissues for eggs to be successfully hatched [8]. Nevertheless, high level RH has been associated with the increase of pathogens and fungi attack [44]. This is further supported by Sajap and Siburat [15], where 90% of fungal infected of *Pteroma pendula* was found on the bottom and middle of tree canopies where the RH was higher than the top canopies. RH, on both extreme ends, could hinder this process through desiccation or drowning of insect eggs [8–11]. Since RH been associated in successfully assisting the breed, grow, and dispersion of certain insects, having the right level of humidity is vital for their survivability. However, as the relationship between the presence of bagworm and RH is nonlinear, the optimum RH values for infestations to occur could not be determined. Over and above that, there has been no study conducted specifically to determine the optimum values of relative humidity for *Metisa plana*.

Despite the other multiple time lags models especially T4, T5 and T6 being lower in accuracies in comparison to the T1, T2 and T3 model, the adjusted R^2^ and error terms were comparable between those two. This observation suggests that this model could be used to predict possible outbreak in an earlier manner so that proper mitigation actions could be taken and the risk could be reduced.

## Conclusions

In this study that focuses on quantifying the relationship between RH and *Metisa plana*’s infestations, it was found that the ANN was better in capturing the relationship between the abiotic factor i.e. RH and the presence of *Metisa plana* when compared to the regression analysis. The ANN algorithm in combination with remotely sensed derived RH was able to predict the presence of *Metisa plana* three weeks before an outbreak possibly occur with 95.29% accuracy. This geospatial model would allow for an early warning system to be developed. Such a system would assist the decision making process by plantation’s managements to control *Metisa plana*’s outbreak by providing them with a quantitative information on the potential magnitude of *Metisa plan*a’s infestations. Consequently, mitigation actions could be taken at their earliest at specific potential locations, and thus, permit the plantation management to strategize for the most efficient controlling method that would save cost, man power, and time.

Finally, while the study only focused on a single abiotic factor that might influence the *Metisa plana*’s population i.e. RH, it is known that insects’ population are significantly impacted by other factors such as temperature and rainfall. Future works should include the assessment of calibrated remotely sensed temperature and rainfall data for predicting population of *Metisa plana*, specifically or other insect pests, generally.
